# The application of single-hole laparoscopic repair with slow-absorbable suture in the treatment of indirect inguinal hernia in children

**DOI:** 10.1007/s10029-026-03608-8

**Published:** 2026-03-16

**Authors:** Chenyao Wang, Yuelan Zheng, Qi Feng

**Affiliations:** Department of Pediatric Surgery, Shenzhen Children’s Hospital, Shenzhen, China

**Keywords:** Indirect inguinal hernia, Laparoscope, Slow-absorbable suture, Non-absorbable suture

## Abstract

**Background:**

The choice of suture material for laparoscopic high ligation of pediatric indirect inguinal hernia remains debated. While non-absorbable sutures are traditionally preferred to minimize recurrence, they pose potential long-term risks as permanent foreign bodies, including suture reaction and theoretical oncogenic concerns. Recent expert consensus suggests absorbable sutures may be a viable alternative, though comparative evidence, particularly for slow-absorbable variants, is limited.

**Objective:**

This study aimed to compare the surgical outcomes, specifically recurrence rates and suture-related complications, between slow-absorbable (Polydioxanone, PDS) and non-absorbable (MERSILK) sutures in single-port laparoscopic indirect inguinal hernia repair in children.

**Methods:**

A retrospective analysis was conducted on 1022 children with unilateral indirect inguinal hernia who underwent surgery at our center between October 2022 and October 2023. Patients were divided into two groups based on the suture material used: a slow-absorbable suture group (*n* = 663) and a non-absorbable suture group (*n* = 359). Patient demographics, operative details, and postoperative complications (recurrence and suture knot reaction) were compared. Univariate and multivariate logistic regression analyses were performed to identify independent risk factors for recurrence.

**Results:**

The recurrence rate was 2.0% (13/663) in the slow-absorbable suture group and 0.8% (3/359) in the non-absorbable suture group; this difference was not statistically significant (*P* = 0.197). Multivariate analysis confirmed that suture type was not an independent risk factor for recurrence [OR = 1.898, 95% CI: 0.475–7.583, *P* = 0.365]. In contrast, a statistically significant higher incidence of suture knot reaction was observed in the non-absorbable group (0.8% vs. 0%, *P* = 0.043). Patient age and the internal ring diameter of theprocessus vaginalis were identified as significant independent risk factors for recurrence (*P* < 0.001 and *P* = 0.001, respectively).

**Conclusion:**

The use of slow-absorbable sutures (PDS) for laparoscopic indirect inguinal hernia repair in children does not significantly increase recurrence rates compared to non-absorbable sutures (MERSILK), while effectively eliminating the risk of suture knot reactions. Recurrence is primarily determined by patient age and internal ring diameter, not suture absorbability. Slow-absorbable sutures present a safe and effective alternative, alleviating long-term foreign body concerns without compromising surgical success.

## Introduction

**Fig. 1:**
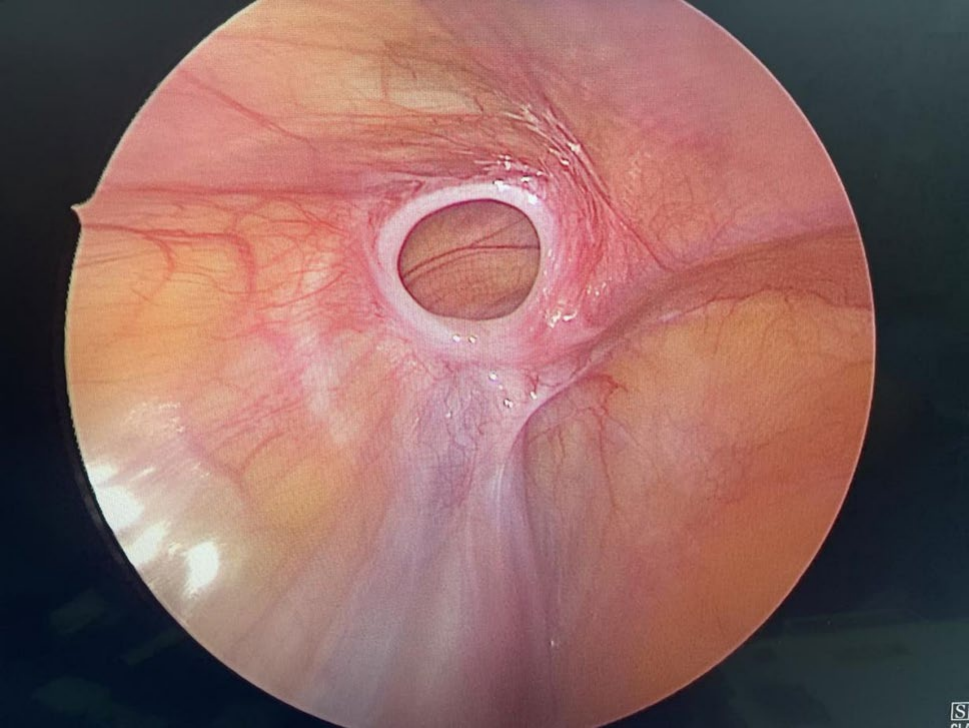
The second surgical exploration of the slow-absorbable suture material group showed that the suture around the internal ring of the processus vaginalis had been completely absorbed

**Fig. 2:**
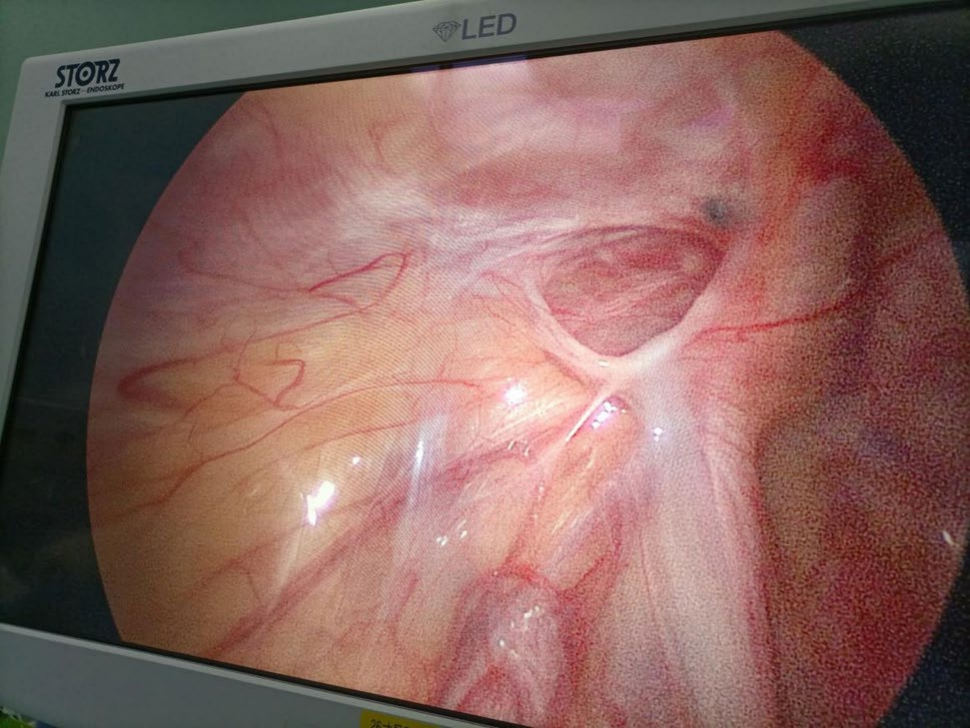
In the non-absorbable suture material group, the second surgical exploration showed that the non-absorbable suture still remained around the open internal ring of the processus vaginalis

**Fig. 3:**
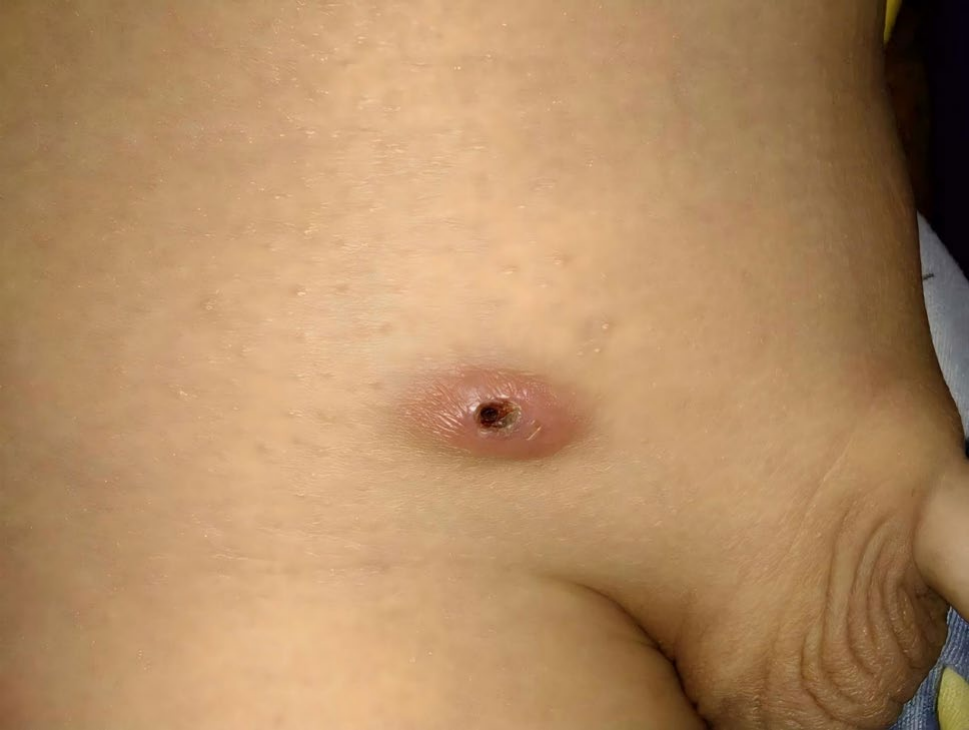
Three weeks after the operation, an inflammatory mass formed due to suture reaction at the internal ring puncture site

**Fig. 4:**
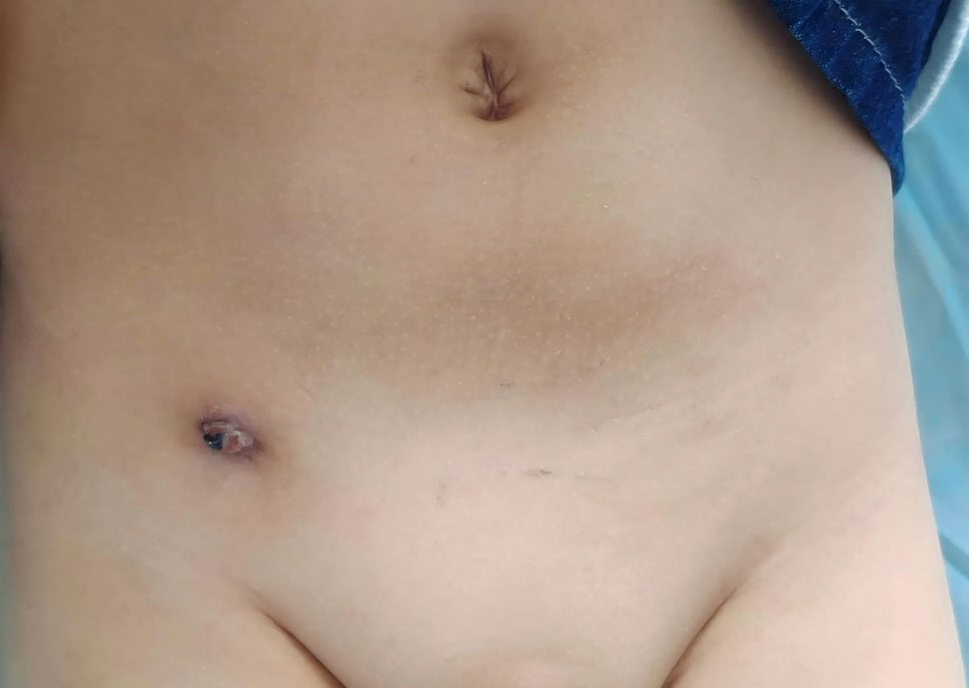
Six weeks after the operation, the inflammatory mass at the internal ring puncture site ulcerated, and the suture knots were exposed

**Fig. 5:**
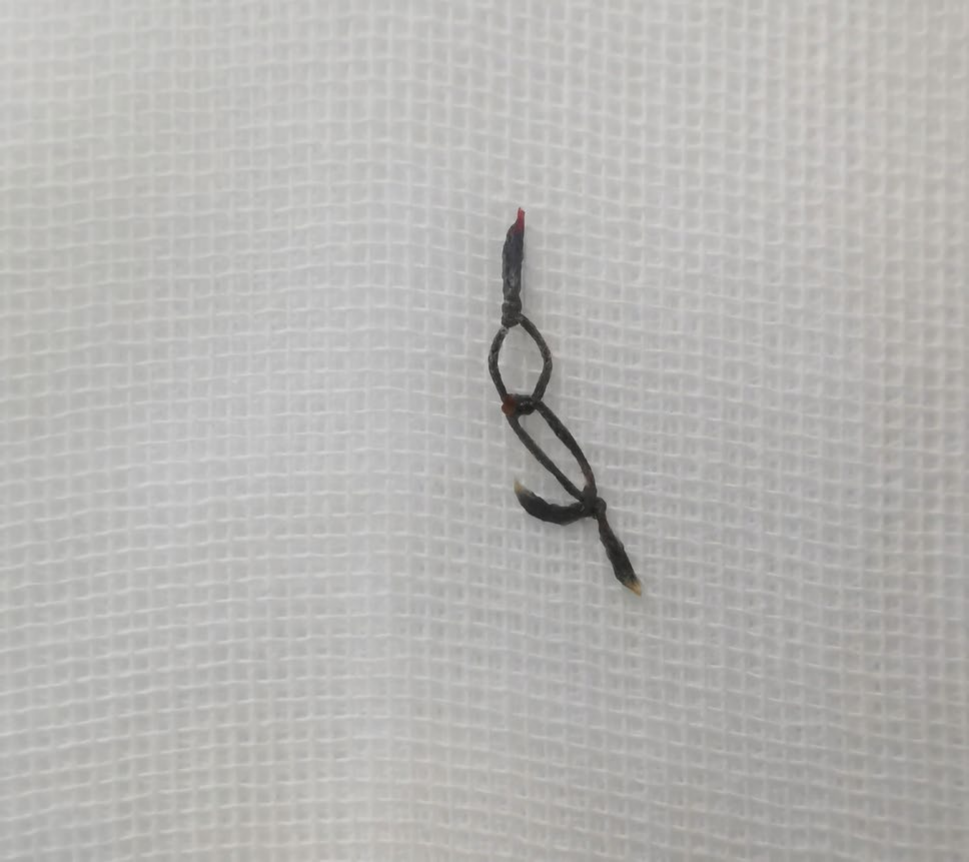
Twelve weeks after the operation, the knot of the sutures was cut and removed

Indirect inguinal hernia is a common malformation in pediatric surgery, which refers to occlusion pause, delay, incomplete occlusion or complete patent processus vaginalis (Nück canal) [1]. At birth, the open state of the sphingoid process was observed in 80% of neonates, which decreased significantly after 6 months and reached a plateau after 3 to 5 years. Indirect inguinal hernia was more common in males, with a male to female ratio of 3:1 to 10:1. The principle of treatment for indirect inguinal hernia in children is high ligation of the processus vaginalis. In recent years, with the improvement of laparoscopic surgical skills and instruments, laparoscopic treatment of children with indirect inguinal hernia has gradually emerged and tends to replace traditional surgery [2, 3]. In consideration of the firm ligation of the internal ring and the long-term reduction of the recurrence rate, the traditional custom at home and abroad is to use non-absorbable suture ligation or suture of the internal ring, but the non-absorbable suture is permanently retained in the human body as a foreign body, and a small number of children will have non-absorbable suture rejection and need to be removed again, and even may have the risk of malignant transformation in the long term [4–7]. Moreover, long-term effects on fertility cannot be completely excluded [8–11]. In 2021, the Chinese Expert consensus on Suture Techniques and Materials for Laparoscopic hernia and Abdominal Wall Surgery released by China stated that absorbable sutures such as polydioxanone sutures (such as PDS Plus) can be used in pediatric inguinal hernia. Glycan-lactic acid suture (such as Vicryl Plus) or non-absorbable suture (such as Ethibond) for hernia sac ligation [12]. Based on the current research progress and the above expert consensus. This study was carried out in our center to provide clinical evidence for the application of slowabsorbable suture such as PDS in the surgical treatment of pediatric indirect inguinal hernia. The aim of this study is to analyze and summarize the surgical process and the occurrence of intraoperative and postoperative complications in children who underwent single-port laparoscopic internal ring ligation with absorbable suture (absorbable suture group) and non-absorbable suture (non-absorbable suture group) in our center Figs. 1, 2, 3, 4, and 5.

## Information and methods

### General information

The clinical data of 1022 cases with unilateral indirect inguinal hernia who underwent single-port laparoscopic-assisted surgery with absorbable suture and non-absorbable suture in Shenzhen Children's Hospital from October 2022 to October 2023 were collected. Inclusion criteria: The children were diagnosed with unilateral indirect inguinal hernia, and there were no obvious surgical contraindications. Exclusion criteria: incarcerated hernia, preoperative or existing cases of testicular dysplasia on the affected side, and other underlying diseases. The surgical methods and the characteristics, advantages and disadvantages of these two surgical materials were explained to the parents before the operation, and they were free to choose, and signed the informed consent form for the operation. The patients were divided into two groups according to different suture materials: slowabsorbable suture group (663 cases) and non-absorbable suture group (359 cases).

### Methods

#### Suture introduction

1. Slow-absorbable suture PDS: This suture is produced by Johnson & Johnson (Shanghai) Medical Equipment Co., LTD., registration No.: Guokao Injection 20153653327. PDS™ II is a sterile synthetic absorbable single-fiber surgical suture made of polyester (polydioxanone). The polydioxanone polymer is non-antigenic and non-thermogenic, producing only a slight tissue reaction during absorption. PDS™ II sutures cause mild inflammation at the initial stage of suture, which is eventually replaced by inward growing fibrous connective tissue. Due to hydrolysis, PDS™ II sutures will gradually lose their tensile strength and will eventually be absorbed. "The polymer is degraded to a monomeric acid (2-hydroxyethoxy) acetic acid, which is then taken up and excreted." The absorption process begins with a decrease in tension strength followed by the absorption of the suture material. The mouse implantation study showed that the residual tension strength of PDS M 2.0 (3–0) and thicker sutureswas 80% of the original at 14 days, 70% at 28 days, and 60% at 42 days after implantation. This suture is particularly suitable for sites where both suture absorption and prolonged wound support are required.
2. Non-absorbable suture MERSILK: This suture is produced by Johnson & Johnson (Shanghai) Medical Equipment Co., LTD., registration No.: 20202020196. Johnson & Johnson Silk Knitted Non-absorbable suture is made by a silk knitting process, which gives it high strength and durability. This technique enables the suture to withstand greater tension and is not easy to break, thus ensuring the firmness and reliability of the surgical suture. With good biocompatibility and corrosion resistance, this material does not cause allergic reactions and is non-irritating to human tissues, thus reducing the risk of postoperative complications.

#### Surgical methods

All the children were treated with general anesthesia, and the skin and subcutaneous tissue were cut through a 0.5 cm arc-shaped incision at the lower edge of the umbilical cord. A pneumoperitoneum needle was inserted, carbon dioxide gas was injected, artificial pneumoperitoneum was established, a 5 mm Trocar was placed, and a 30° laparoscopy was placed. A 2 mm incision was made at the surface of the inner ring on the affected side, and the hernia needle was penetrated vertically through the incision. 2–0 PDS or MERSILK thread was used to penetrate through the inner half of the inner ring outside the peritoneum. Tighten the suture and knot to close the inner ring (ligation twice). There was no bleeding in the abdominal cavity, no injury of blood vessels and intestinal vas deferens. The fascia layer was sutured with absorbable suture, the skin wounds were bonded with medical glue, and the dressings were applied.

#### Observation indicators and statistical analysis

The gender, age, percentage of physical growth (The specific grouping was as follows: BMI < P3 was defined as lean body type; P3 ≤ BMI < P85 was defined as normal body size; P85 ≤ BMI < P97 was defined as overweight. BMI ≥ P97 was defined as obese body type.), operation time, length of hospital stays, follow-up time and diameter of the inner ring of the processus vaginalis, and postoperative complications of the two groups were recorded in detail. Indicators for evaluating the suture reaction: Clinical symptoms include redness, swelling, a localized subcutaneous mass, and ulceration at the abdominal wall puncture site of the internal ring. Ultrasonography revealed subcutaneous effusion at the lesion site, within which a moderately echogenic focus was visualized, suggestive of a foreign body. SPSS 27.0 was used for statistical analysis. Normally distributed continuous variables are described using‾x ± s, while non-normally distributed variables are presented as median (interquartile range). Categorical variables are expressed as percentages (%) with absolute numbers (n). Differences in continuous variables were tested using Student's t-test or Wilcoxon rank-sum test. Differences in categorical variables were analyzed using chi-square test with dummy variables. A *P*-value < 0.05 was considered statistically significant [13]. Univariate logistic regression analysis was performed for age, gender, physical growth percentiles, operative time, hospital stay, follow-up duration, and internal ring diameter of processus vaginalis. Variables with *P* < 0.05 were identified as potential independent risk factors for postoperative recurrence rates.

## Result

### Comparison of patient baseline characteristics

A total of 1022 patients were enrolled in this study, comprising 663 in the slow-absorbable suture material group and 359 in the non-absorbable suture material group. All enrolled patients had unilateral indirect inguinal hernia. A comparison of the baseline characteristics between these two groups is detailed in Table 1. The results showed no statistically significant differences between these two groups regarding age, operative time, or internal ring diameter of the processus vaginalis (*P* > 0.05), indicating comparability between the groups for these baseline indicators. However, significant differences were observed in the distribution of gender, hospitalization time, follow-up time, and physical growth percentile (BMI) (*P* < 0.05). Specifically, the non-absorbable suture material group had a higher proportion of male patients (75.5% vs. 68.6%) and a significantly higher proportion of patients with a BMI below the 3rd percentile (P3) (68.8% vs. 49.9%). These differences were identified as potential confounding factors and were thus controlled for in the subsequent multivariate analysis.

**Table 1:** Baseline Characteristics of the Groups

Variable	Slow-absorbable suture material group	Non-absorbable suture material group	statistical magnitude	*P-Value*
Age (years)	**3 (2, 5)**	**4 (2, 5)**	***Z* = −0.74**	**0.460**
Gender:			***χ***^**2**^** = 5.327**	**0.021**
Male	**455 (68.6%)**	**271 (75.5%)**		
Female	**208 (31.4%)**	**88 (24.5%)**		
Hospitalization time (days)	**1 (1, 3)**	**1 (1, 3)**	***Z* = −6.54**	** < 0.001**
Follow-up time (months)	**6 (5, 6)**	**5 (5, 7)**	***Z* = −0.73**	** < 0.001**
Operative time (minutes)	**12 (10, 15)**	**12 (10, 15)**	***Z* = −1.13**	**0.258**
Internal ring diameter of the processus vaginalis (cm)	**1.4 (1.2, 1.6)**	**1.4 (1.2, 1.6)**	***Z* = −0.09**	**0.928**
Physical growth percentile:			***Z* = −4.39**	** < 0.001**
BMI < P3	**331 (49.9%)**	**247 (68.8%)**		
P3 ≤ BMI < P85	**249 (37.6%)**	**56 (15.6%)**		
P85 ≤ BMI < P97	**64 (9.7%)**	**38 (10.6%)**		
P97 ≤ BMI	**19 (2.9%)**	**18 (5.0%)**		

### Univariate comparison of outcome indicators

A comparison of postoperative outcome indicators between two groups is presented in Table 2. Regarding the primary outcome indicator, recurrence rate, the slow-absorbable suture material group had a recurrence rate of 2.0% (13/663), compared to 0.8% (3/359) in the non-absorbable suture material group. Although the recurrence rate was numerically higher in the slow-absorbable suture group, the intergroup difference did not reach statistical significance (*P* = 0.197). For the secondary outcome indicator, suture knot reaction, the incidence in the non-absorbable suture material group was 0.8% (3/359), whereas no suture knot reactions (0%) were observed in the slow-absorbable suture material group. This difference was statistically significant (*P* = 0.043).

**Table 2:** Comparison of the outcome indicators between two groups of patients

Variable	Slow-absorbable suture material group	Non-absorbable suture material group	statistical magnitude	*P-Value*
Recurrence	**13 (2.0%)**	**3 (0.8%)**	**-**	**0.197**
Suture knot reaction	**0 (0%)**	**3 (0.8%)**	**-**	**0.043**

### Logistic regression analysis of risk factors for recurrence

To control for the potential influence of baseline imbalances and to identify independent risk factors for recurrence, a binary logistic regression analysis was performed. Prior to the regression analysis, collinearity diagnostics (Table 3) confirmed that the Variance Inflation Factor (VIF) for all independent variables was well below 10 (maximum value = 1.448), indicating the absence of severe multicollinearity and supporting the reliability of the analysis results. The logistic regression analysis results (Table 4) revealed the following: Suture type was not an independent risk factor for hernia recurrence [OR = 1.898, 95% CI: (0.475, 7.583), *P* = 0.365]. Patient age [OR = 1.393, 95% CI: (1.164, 1.668), *P* < 0.001] and internal ring diameter of the processus vaginalis [OR = 258.444, 95% CI: (9.700, 6885.558), *P* = 0.001] were identified as independent risk factors for recurrence. Each one-year increase in age was associated with an approximately 39.3% higher risk of recurrence, while an increase in the internal ring diameter was associated with a sharp rise in recurrence risk. Gender, operative time, hospitalization time, and physical growth percentile (BMI categories) showed no significant association with recurrence risk in this model (*P* > 0.05).

**Table 3:** Collinearity analysis

	Unstandardized coefficient		Standardized coefficient	t	significance	Collinearity statistics	
	B	standard error	Beta			Tolerance Interval	VIF
constant (quantity)	**0.027**	**0.032**		**0.846**	**0.398**		
Type of Suture	**−0.011**	**0.008**	**−0.042**	**−1.366**	**0.172**	**0.948**	**1.055**
Gender	**0.018**	**0.008**	**0.066**	**2.144**	**0.032**	**0.941**	**1.063**
Age	**0.006**	**0.002**	**0.131**	**4.204**	** < 0.001**	**0.914**	**1.094**
Physical growth percentile	**0.027**	**0.005**	**0.174**	**4.86**	** < 0.001**	**0.692**	**1.445**
Hospitalization time (days)	**−0.004**	**0.004**	**−0.028**	**−0.91**	**0.363**	**0.927**	**1.079**
Follow-up time (months)	**−0.024**	**0.003**	**−0.276**	**−7.685**	** < 0.001**	**0.691**	**1.448**
Internal ring diameter of the processus vaginalis (cm)	**0.056**	**0.014**	**0.116**	**3.877**	** < 0.001**	**0.992**	**1.008**
Operative time (minutes)	**0.001**	**0.001**	**0.027**	**0.889**	**0.374**	**0.992**	**1.008**

Dependent variable:Recurrence

**Table 4:** Binary Logistic Regression: Analysis of Risk Factors for Recurrence

Variable	*β*	SE	Exp(B)	95%CI		*P*-Value
				Lower limit	Upper limit	
Type of Suture	**0.641**	**0.707**	**1.898**	**0.475**	**7.583**	**0.365**
Age	**0.332**	**0.092**	**1.393**	**1.164**	**1.668**	** < 0.001**
Gender	**−1.416**	**0.797**	**0.243**	**0.051**	**1.158**	**0.076**
Internal ring diameter of the processus vaginalis (cm)	**5.555**	**1.675**	**258.444**	**9.7**	**6885.558**	**0.001**
Operative time (minutes)	**0.052**	**0.066**	**1.054**	**0.926**	**1.199**	**0.426**
Hospitalization time (days)	**−0.669**	**0.422**	**0.512**	**0.224**	**1.171**	**0.113**
BMI < P3						**0.859**
P3 ≤ BMI** < **P85	**−0.959**	**1.181**	**0.383**	**0.038**	**3.879**	**0.417**
P85 ≤ BMI** < **P97	**−0.665**	**1.217**	**0.514**	**0.047**	**5.579**	**0.584**
P97 ≤ BMI	**−0.802**	**1.507**	**0.448**	**0.023**	**8.606**	**0.595**

## Discussion

In recent years, the choice of suture material has emerged as a primary point of contention in minimally invasive laparoscopic high ligation of the indirect inguinal hernia in children [14–16]. The prevailing preference among many pediatric surgeons has been the use of non-absorbable sutures, aiming to minimize the risk of postoperative recurrence. However, this practice raises two significant concerns. Firstly, non-absorbable sutures, as permanent foreign bodies, reside in proximity to critical structures like the spermatic cord and vas deferens, potentially inciting chronic inflammation that could impair their long-term function. Secondly, persistent apprehensions exist regarding the long-term oncogenic risk of implanted surgical materials. Studies have indicated that various polymers, including polyester, nylon, and silk, can induce foreign body carcinogenesis [4–8]. Supporting this, Birolini et al. reported cases of abdominal wall squamous cell carcinoma arising at the site of mesh implants following chronic infection, highlighting a potential, albeit rare, serious complication [6].

Driven by these considerations, the use of absorbable sutures in pediatric indirect inguinal hernia repair has been explored. Early work by Felix Schier et al. in 2002, in a multi-center retrospective study, found a recurrence rate of 2.9% for a surgeon using absorbable sutures, comparable to rates of 3.4–3.5% for surgeons using non-absorbable sutures, concluding that suture absorbability was not a determining factor for recurrence [17]. This finding was corroborated by Ozgediz et al., who, in 2007, found no significant difference in recurrence rates between absorbable (5%) and non-absorbable (3.8%) sutures [18]. Conversely, other series reported higher recurrence rates with absorbable sutures (4.8%) compared to very low rates (0.5–0.9%) with non-absorbable ones, leading Bharathi et al. to hypothesize that premature absorption before complete sac healing might be the cause [19]. A more recent study by G.M. Grimsby et al. in 2015 strongly favored non-absorbable sutures, reporting a significantly higher recurrence rate with short-acting absorbable polyglactin (26%) versus non-absorbable sutures (4%), attributing this to the insufficient duration of tensile support [20]. This conflicting evidence underscores the ongoing debate regarding the suitability of absorbable sutures, particularly slow-absorbable variants, for pediatric indirect inguinal hernia repair, with a notable lack of focused studies on this specific suture type [12, 21].

Our study directly addresses this gap by comparing slow-absorbable and non-absorbable suture materials. The initial univariate analysis (Table 2) showed no statistically significant difference in recurrence rates between the slow-absorbable (2.0%) and non-absorbable (0.8%) groups (*P* = 0.197). Crucially, this finding was confirmed in the multivariate binary logistic regression analysis (Table 4), which controlled for identified baseline imbalances (Table 1) and potential confounders. The regression model, validated for the absence of multicollinearity (Table 3), demonstrated that the type of suture was not an independent risk factor for recurrence [OR = 1.898, 95% CI: 0.475–7.583, *P* = 0.365]. This central finding aligns with the conclusions of Schier [17] and Ozgediz [18] and supports the conditional recommendation for absorbable suture use in the 2021 Chinese Expert Consensus, for which our study now provides substantive evidence.

Importantly, our study offers a critical insight that may help reconcile the conflicting literature: the type of absorbable suture is paramount. The high recurrence rate (26%) reported by Grimsby et al. [20] was associated with short-acting polyglactin. In our cohort using slow-absorbable sutures, the recurrence rate was markedly lower. This suggests that slow-absorbable sutures provide adequate mechanical support throughout the critical healing period, mitigating the risk of premature failure. This is further substantiated by our observation that recurrences in the slow-absorbable group occurred between 1 and 4 months postoperatively, during which surgical re-exploration confirmed the sutures had been completely absorbed, indicating that the healing process was either incomplete or insufficiently robust at the time of absorption (Fig.1). However, non-absorbable suture group remained intact (Fig.2). 

Beyond recurrence, a significant advantage of slow-absorbable sutures was identified in the reduction of suture-related complications. We observed a statistically significant higher incidence of suture knot reactions in the non-absorbable group (0.8%) compared to a complete absence (0%) in the slow-absorbable group (*P* = 0.043, Table 2). 

In non-absorbable suture group, it was clinically observed that at three weeks postoperatively, an inflammatory mass typically formed at the internal ring puncture site due to suture knot reaction(Fig.3). At approximately six weeks postoperatively, the inflammatory mass at the internal ring puncture site usually ulcerated(Fig.4). In these cases of suture knot reaction, surgical removal of the suture was generally performed at twelve weeks after the initial surgery(Fig.5).This finding directly addresses one of the theoretical concerns regarding permanent sutures and suggests that the use of slow-absorbable materials can effectively eliminate this specific complication, thereby potentially improving patient comfort and reducing long-term foreign body risks.

Finally, our logistic regression analysis (Table 4) identified patient age and internal ring diameter as powerful, independent risk factors for recurrence, consistent with established surgical principles. This underscores that technical success and long-term outcomes depend not only on the choice of suture material but also critically on patient-specific anatomical and physiological factors.

## Limitations

The single-center design may limit the generalizability of our findings. Therefore, large-scale, prospective, randomized controlled trials are warranted to validate these results and provide a higher level of evidence.

## Conclusion

In conclusion, our study demonstrates that the use of slow-absorbable sutures for laparoscopic high ligation of the indirect inguinal hernia in children does not lead to a statistically significant increase in recurrence rates compared to non-absorbable sutures, while simultaneously eliminating the risk of suture knot reactions. The key determinants of recurrence are patient age and the internal ring diameter, not the absorbability of the suture material. We propose that slow-absorbable sutures represent a safe and effective alternative for pediatric indirect inguinal hernia repair, mitigating long-term foreign body concerns without compromising surgical efficacy.

## Data Availability

Participants were assured that their identities would remain confidential, and any identifying information would be anonymized prior to publication. All participants were informed that their data would be used for research purposes and published in a scientific journal. After publication, all data materials will be available in a public database.
